# Protocol for circular dichroism spectral analysis of the thermal stability of CpG-methylated quadruplex structures

**DOI:** 10.1016/j.xpro.2025.103646

**Published:** 2025-02-21

**Authors:** Momo Moriya, Taiji Oyama, Masanori Goto, Kazunori Ikebukuro, Wataru Yoshida

**Affiliations:** 1 Graduate School of Bionics, Tokyo University of Technology, 1404-1 Katakura, Hachioji, Tokyo 192-0982, Japan; 2 Department of Biotechnology and Life Science, Tokyo University of Agriculture and Technology, 2-24-16 Naka-cho, Koganei, Tokyo 184-8588, Japan; 3 Sales Division, JASCO Corporation, 2967-5 Ishikawa, Hachioji, Tokyo 192-8537, Japan; 4 School of Bioscience and Biotechnology, Tokyo University of Technology, 1404-1 Katakura, Hachioji, Tokyo 192-0982, Japan

**Keywords:** bioinformatics, circular dichroism, CD, molecular biology

## Abstract

G-quadruplex and intercalated motif are quadruplex structures of which sequences are enriched in promoters. Here, we present a protocol for circular dichroism spectral analysis of the thermal stability of CpG-methylated quadruplex structures. We describe steps for preparing the oligonucleotide sample, measuring the circular dichroism spectrum of methylated quadruplex structures, and calculating thermodynamic parameters using Python 3.

For complete details on the use and execution of this protocol, please refer to Kimura et al.^1^

## Highlights

• Preparation of CpG-methylated quadruplex-forming oligonucleotides
• Circular dichroism spectroscopy of the quadruplex structure
• Calculation of the thermodynamic parameters by Python 3

## Abstract (editor highlights)

Publisher’s note: Undertaking any experimental protocol requires adherence to local institutional guidelines for laboratory safety and ethics.

## Graphical abstract

**Figure:**
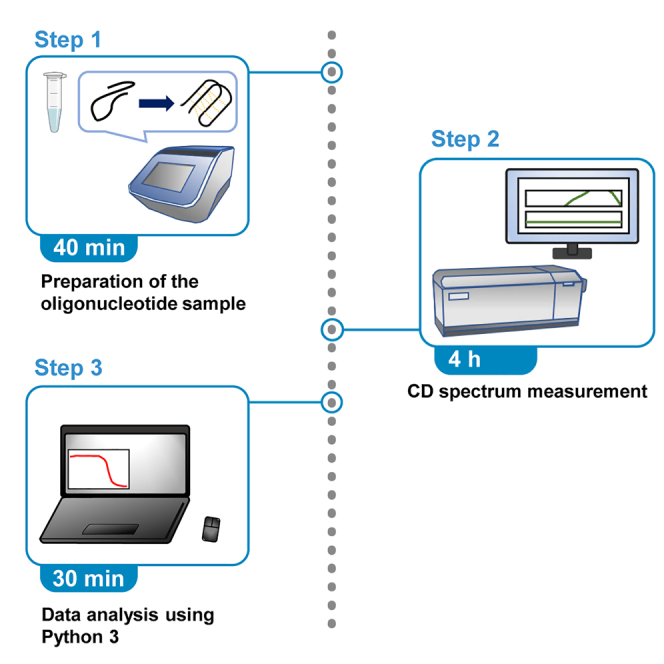

## Before you begin

**Figure 1:**
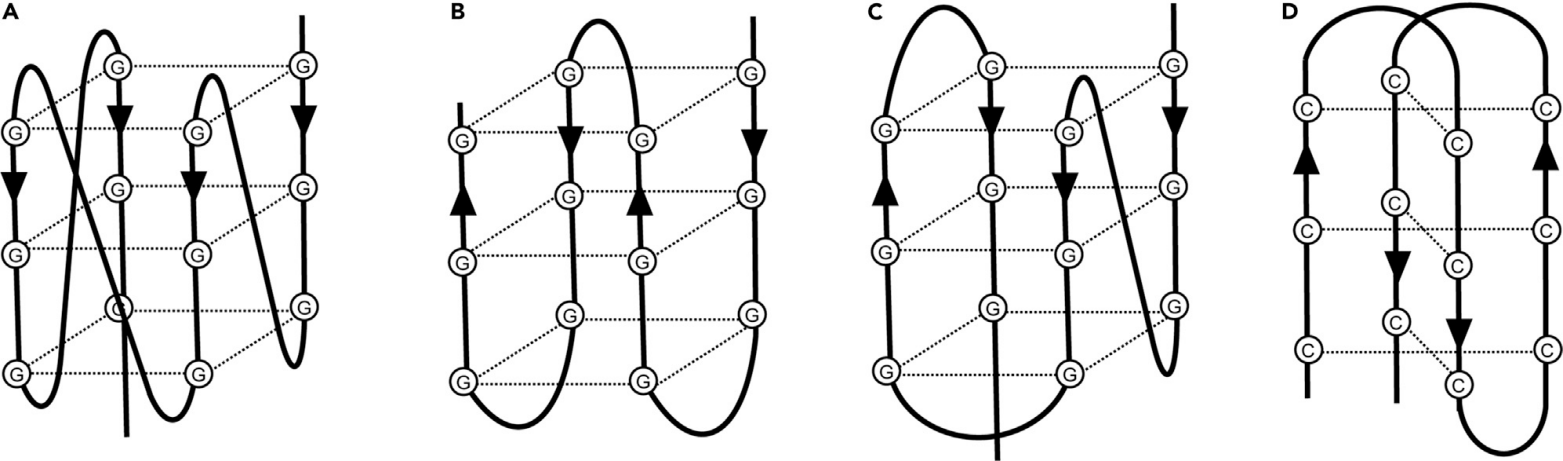
Secondary structures of the quadruplexes (A) Parallel G4; (B) antiparallel G4; (C) hybrid G4; (D) i-motif.

In cells, DNA mainly folds into a double-helical structure formed by Watson–Crick base pairing, but it may also fold into non-canonical DNA structures, such as a triplex, stem-loop, cruciform, G-quadruplex (G4), and intercalated motif (i-motif) (Figure 1).^2^ Among them, G4, the most studied non-canonical structure, is formed by the stacking of two or more G-quartets composed of four guanine bases connected by Hoogsteen hydrogen bonds. In genomic DNA, G4 structures are formed in G-rich strands and are involved in transcription,^3^^,^^4^^,^^5^ translation,^6^ splicing,^7^ replication,^8^ and telomere maintenance.^9^ *In silico* and *in vitro* approaches have identified numerous G4-forming sequences in cis-regulatory elements such as CpG islands and promoters.^10^^,^^11^^,^^12^^,^^13^^,^^14^ In contrast, i-motif structures may form in C-rich strands that are complementary to G4-forming sequences, and is involved in transcriptional regulation.^15^^,^^16^

G4 structures are stabilized by monovalent cations, especially potassium ions, coordinated between G-quartets. I-motif structures are formed under acidic conditions because hemiprotonated cytosine base pairs (C–C^+^) are required for folding into the i-motif structure. In addition to the solution conditions, CpG methylation affects the stability of G4 and i-motif structures. The methyl group is an electron donor; therefore, 5-methylcytosine increases the molecular polarizability of pyrimidine, resulting in enhanced stacking interactions.^17^^,^^18^ CpG methylation of G4 structures affects the binding activity of target proteins.^19^ Methylation also increases the base-pairing energy of C–C^+^.^20^^,^^21^ Moreover, N^6^-methyladenine (m^6^A) modifications affect G4 structures because m^6^A also enhances stacking interactions and inhibits the formation of hydrogen bonds at the N^6^ position.^22^^,^^23^^,^^24^ The analysis of the effect of epigenetic modifications on G4 and i-motif structures is important for elucidating the biological role of epigenetic modifications in G4- and i-motif-forming regions.

**Figure 2:**
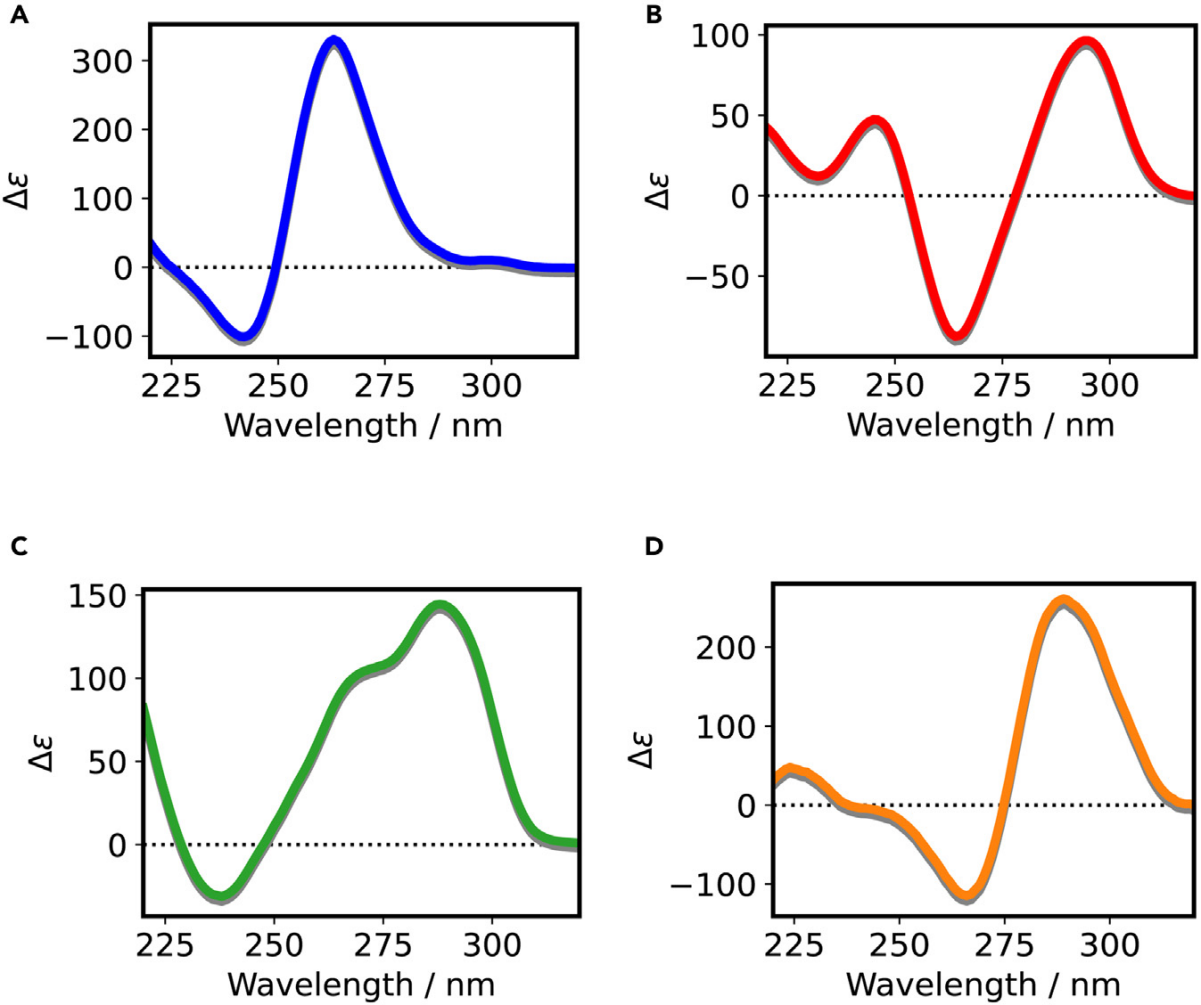
Representative CD spectra of quadruplexes (A) Parallel G4; (B) antiparallel G4; (C) hybrid G4; (D) i-motif.

The topologies of G4 structures are classified as parallel, antiparallel, or hybrid. These G4 structures possess specific circular dichroism (CD) spectra: parallel G4 shows a positive peak at approximately 260 nm and a negative peak at approximately 245 nm; antiparallel G4 shows a positive peak at approximately 295 nm and a negative peak at approximately 260 nm; and hybrid G4 shows two positive peaks at approximately 295 and 260 nm and a negative peak at approximately 245 nm.^25^^,^^26^ The i-motif structure exhibits a specific CD spectrum with a positive peak at approximately 290 nm and a negative peak at approximately 265 nm (Figure 2).^27^ The thermal stability of G4 and i-motif structures can be evaluated by measuring the CD values at the positive peaks from 25°C to 95°C.

**Figure 3:**
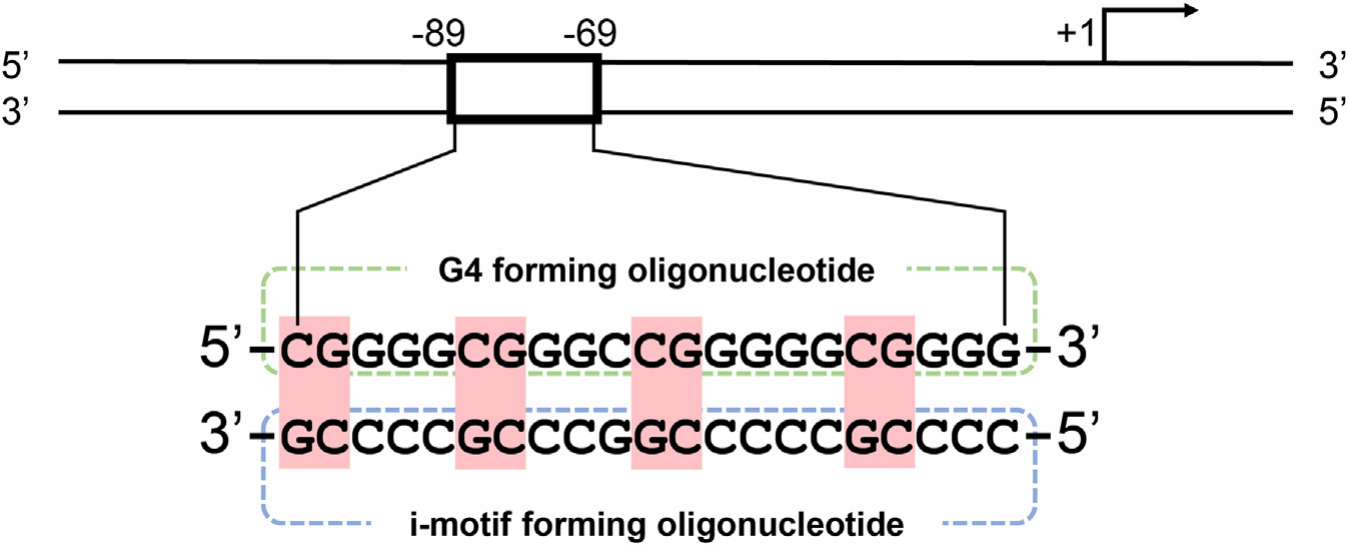
Location of the G4 and i-motif-forming sequences in the *VEGF* gene The top and bottom strands contain the G4 and i-motif-forming sequences, respectively.

In this protocol, a detailed procedure for the measurement of the CD spectrum and calculation of the thermodynamic parameters ($T_{m}$, ΔH, and ΔS) based on Python 3 is described. The protocol below describes the specific steps for using *VEGF* i-motif-forming oligonucleotide, which is complementary to the *VEGF* G4-forming oligonucleotide (Figure 3). However, we have also used this protocol in G4 and other i-motif-forming oligonucleotides of which thermal denaturation obeys two-state thermal denaturation.

### Synthesis of oligonucleotides

**Timing: <3 weeks**

CpG-methylated (5-methylcytocyine modified) oligonucleotides can be purchased from various companies; however, unlike unmodified oligonucleotides, it takes several weeks to obtain them. In this protocol, lyophilized oligonucleotides obtained from the Tsukuba Oligo Service were used, which took 15 days to obtain. The oligonucleotides should be purified by high-performance liquid chromatography (HPLC) because byproducts affect the CD spectrum. The sequences of the unmethylated and methylated CpG *VEGF* i-motif-forming oligonucleotides are listed in key resources table.

**Figure 4:**
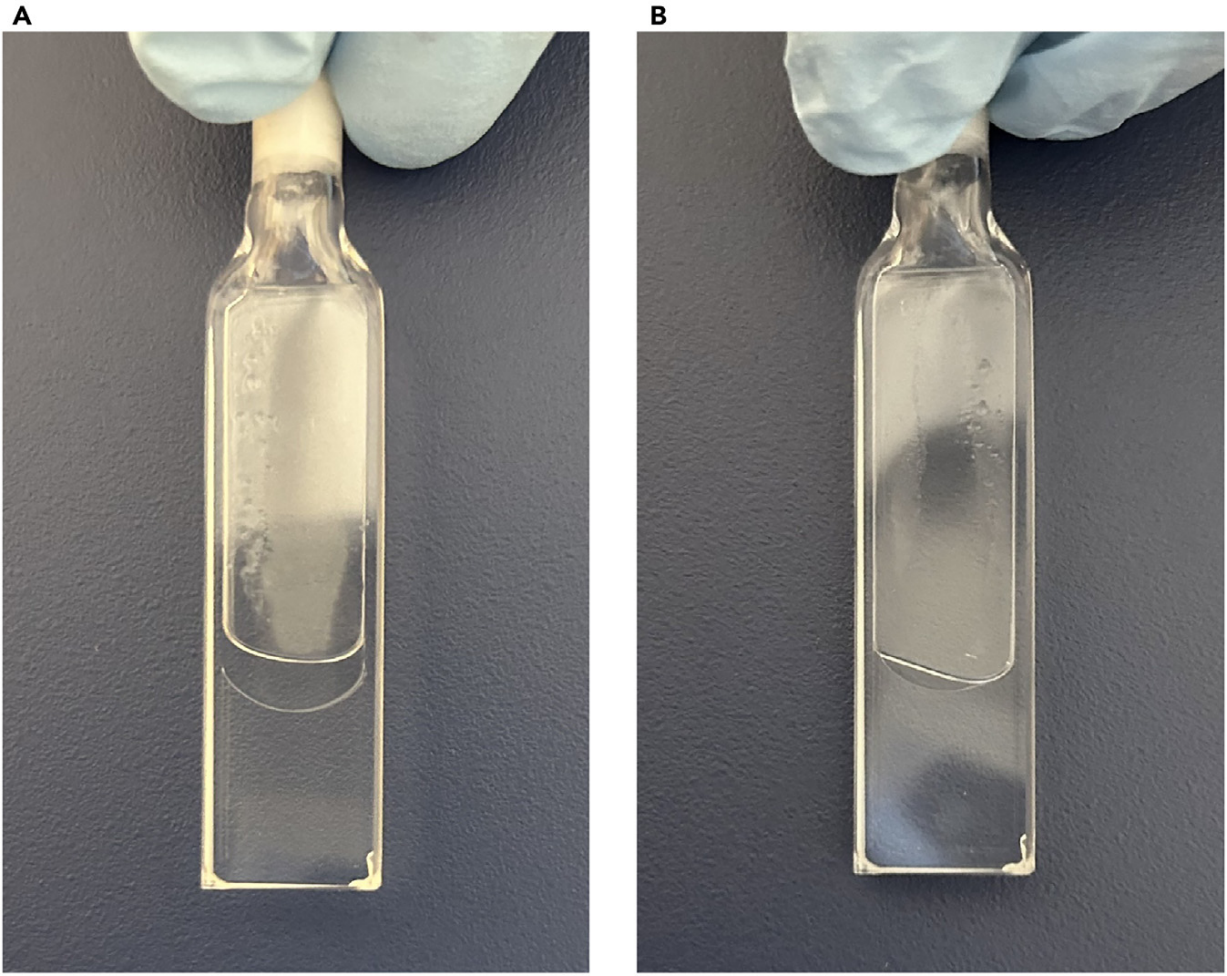
Cuvette with oligonucleotide sample and mineral oil The top surface of the sample is covered (A) or not covered (B) with mineral oil.

## Key resources table

**Table:** 

REAGENT or RESOURCE	SOURCE	IDENTIFIER
**Chemicals, peptides, and recombinant proteins**		

Nitrogen gas	Tomoe Shokai	N/A
Sodium cacodylate	Nacalai Tesque	Cat# 10317-44
Mineral oil, biotechnology grade	Bio-Rad	Cat# 1632129
Hydrochloric acid	Nacalai Tesque	Cat# 18321-05
UltraPure DNase/RNase-free distilled water	Thermo Fisher Scientific	Cat# 10977023
99.5% Ethanol	Nacalai Tesque	Cat# 14741-25

**Oligonucleotides**		

HPLC-purified unmethylated *VEGF* i-motif-forming oligonucleotide: 5′-CCCCGCCCCCG GCCCGCCCCG-3′	Tsukuba Oligo Service	N/A
HPLC-purified methylated *VEGF* i-motif-forming oligonucleotide: 5′-CCC5mCGCCCC5mCG GCC5mCGCCC5mCG-3′ (5mC: 5-methylcytosine)	Tsukuba Oligo Service	N/A

**Software and algorithms**		

Python 3	Python Software Foundation	https://www.python.org

**Other**		

UV/Vis Spectroscopy D30	Eppendorf	N/A
Cuvette with 1 mm optical path length G1.0	Eppendorf	Cat# 6138000018
Veriti Thermal Cycler	Thermo Fisher Scientific	Cat# 4452300
Circular dichroism spectrometer J-1500-450Y	JASCO	N/A
Quartz cell with 1 mm optical path length	JASCO	Cat# 0556
Quartz cell spacer	JASCO	Cat# 7068-1604PA
Mini Diaphragm vacuum pump LABOPORT N 86 kT.18	KNF	N/A

## Materials and equipment

**Table:** Oligonucleotide sample

Reagent	Final concentration	Amount
100 μM oligonucleotide	20 μM	26 μL
250 mM sodium cacodylate buffer (pH 4.8)	25 mM	13 μL
UltraPure DNase/RNase-Free Distilled Water	N/A	91 μL
**Total**	**N/A**	**130 μL**

Store the oligonucleotide stock solution at −20°C and the sodium cacodylate buffer at 22°C–26°C.

***Note:*** For an oligonucleotide of approximately 20 nt, the optimal concentration and optical path length are 20 μM and 1 mm, respectively. If the concentration is changed two or more times, it is recommended that the change the optical path length of the cell be changed, as follows. Ensure that HT does not exceed 600 V because the buffer absorption becomes stronger with longer optical path lengths.

**Table:** 

Concentration [μM]	Optical path length range [mm]
1–4	10
2–10	5
10–40	1
20–100	0.5
100–200	0.1

## Step-by-step method details

### Preparation of the oligonucleotide sample

**Timing: 40 min**

1. Resuspend the lyophilized oligonucleotide in DNase/RNase-free distilled water by vortexing for 5-min to make a stock solution.
2. Dilute the stock solution tenfold with DNase/RNase-free distilled water.
3. Measure the absorbance at 260 nm (A_260nm_) and 320 nm (A_320nm_) of the diluted sample using a spectrometer with a cuvette with 1 mm optical path length.
4. Calculate the oligonucleotide concentration using the following equation.
  a. $\text{Concentration}\;\left(ng/\mu L\right)=\left(A_{260nm}-A_{320nm}\right)\times 10\times 10\times 33$
5. Repeat steps 3–4 twice to calculate the average concentration.
6. Prepare 130 μL of 20 μM oligonucleotide in 25 mM sodium cacodylate buffer, pH 4.8, in a 1.5 mL tube.

***Note:*** For methylated CpG G4 structure analysis, phosphate buffers with adjusted Na^+^ and/or K^+^ concentrations are often used. In the G4 structure, K^+^ is coordinated between the G-quartets, and Na^+^ is coordinated within the G-quartet; therefore, the type and concentration of ions affect the topology and stability of the G4 structures. In the i-motif structure, pH affects formation because protonation of cytosine is required to form C–C^+^. The pH of mid transition of the unmethylated and methylated CpG *VEGF* i-motif-forming oligonucleotides was 6.3 and 6.4, respectively.^1^

7. Transfer 65 μL of the sample to two PCR tubes.
8. Set the PCR tube in a thermal cycler and run the following three steps to fold the i-motif structure.
  a. Incubate at 95°C for 3 min.
  b. Cool the sample to 25°C for 30 min.
  c. Keep the sample at 25°C.

### CD spectrum measurement

**Timing: 4 h**

9. Set the parameters on CD measurement as follows.
  a. Data pitch: 0.1 nm.
  b. Scanning mode: Continuous.
  c. No. of accumulations: 3.
  d. Bandwidth: 1.00 nm.
  e. Scanning speed: 200 nm/min.
  f. Wavelength range: 220 nm–320 nm.
  g. Temperature: from 25°C to 95°C.
  h. Temperature interval: 1°C.
  i. Temperature gradient: 1 °C/min.
10. Add 130 μL of the buffer (25 mM sodium cacodylate buffer, pH 4.8) to the quartz cell.
11. Set the quartz cell on the CD spectrometer and then measure the blank.
12. Discard the buffer. Wash the quartz cell with distilled water twice, followed by washing with 99.5% ethanol once. Dry the quartz cell with a vacuum pump.
13. Add 130 μL of the oligonucleotide sample to the quartz cell, and then add 40 μL of mineral oil on the top of the sample.

**CRITICAL:** Carefully add mineral oil to cover the top surface of the sample (Figure 4A). If the top surface of the sample is not covered with mineral oil (Figure 4B), the sample may evaporate during CD measurement.

14. Set the quartz cell on the CD spectrometer and then measure the CD spectra at wavelengths ranging from 220 nm to 320 nm and at temperatures from 25°C to 95°C increasing at 1°C intervals.
15. Export the data as a csv file.

***Note:*** The csv files contain matrix data for wavelengths in rows and temperatures in columns. In addition to the measurement data, the header and footer contain information about the measurement conditions. The csv files for the unmethylated (VEGF_IM_Um.csv) and methylated *VEGF* i-motifs (VEGF_IM_M.csv ) are provided as supplemental materials.

### Data analysis using Python 3

**Timing: 30 min**

**Figure 5:**
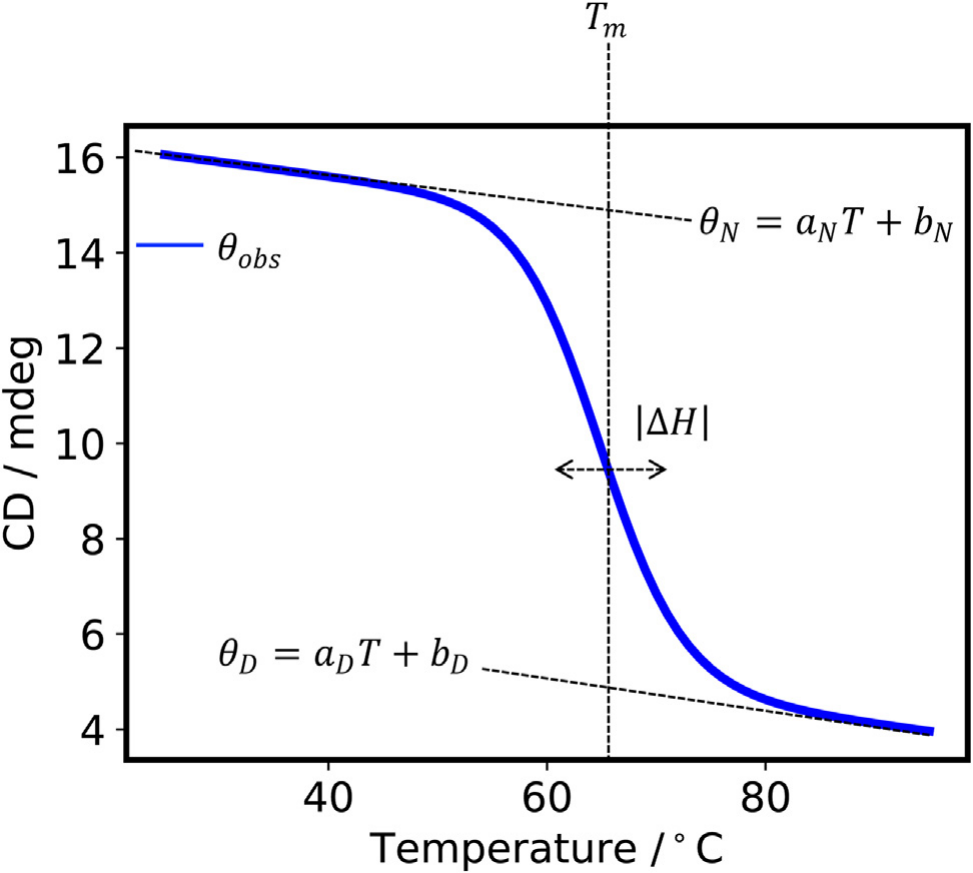
CD melting curve of a two-state thermal denaturation model

The thermodynamic parameters ($T_{m}$, ΔH, ΔS ) of two-state thermal denaturation (N⇋D) were obtained by nonlinear fitting the CD melting curves with Eqs. (Figure 5) (a) through (d), where $\theta_{obs}$ is the measured CD value for a single wavelength as a function of temperature, $\theta_{m}$ (m=N,D) are CD values of N and D defined as linear for temperature, and $K_{N⇋D}$ is the equilibrium constant. This section describes a calculation method using the Levenberg–Marquardt algorithm with SciPy in Python 3.^28^

(a) $\theta_{obs}=\frac{\theta_{m}+{K_{N⇋D}\theta }_{m}}{1+K_{N⇋D}}$, (b) $\theta_{m}=a_{m}T+b_{m}$, (c) $K=\exp\;\left[\frac{\Delta H}{R}\left(\frac{1}{T_{m}}-\frac{1}{T}\right)\right]$, and (d) $\Delta S=\frac{\Delta H}{T_{m}}$

16. Installing Python 3.
  a. Click on [Download Python 3. XX.XX] on the webpage linked below to download the installer.
    https://www.python.org/downloads/.
    ***Note:*** As of December 12, 2024, Python 3 was Python 3.13.1. The present analysis method was performed using Windows.
  b. Check the box “Add Python 3. XX to PATH” and then install Python.
  c. Click [Disable path length limit] and then close the installer.
17. Data analysis using Python 3.
  a. Install the Jupyter Notebook as an integrated development environment. Enter the following command at the command prompt.
    pip install jupyter notebook
  b. Enter the following command at a command prompt to launch the Jupyter Notebook.
    jupyter notebook
  c. When the Jupyter Notebook opens, click in the order of [New] → [Notebook] → [Python 3 (ipykernel)] → [Select].
  d. Execute the following commands one by one to install the library. Commands can be executed by pressing Ctrl + Enter.
    pip install numpy
    pip install scipy
    pip install pandas
    pip install matplotlib
  e. Execute the following code to import the library.
    import pandas as pd
    import numpy as np
    import matplotlib.pyplot as plt
    import scipy.optimize as optimize
  f. Execute the following code to set the graph and font sizes.
    plt.rcParams['font.size']=14
    plt.rcParams['axes.linewidth']=2
  g. Execute the following code to read the CD spectra file.
    VEGF_IM_Um = pd.read_csv(r"Filepass", header=None, skiprows=19, nrows=1001)
    VEGF_IM_Um
    ***Note:*** In this protocol, the name is “VEGF_IM_Um,” but any name can be specified. Enter the file path of the CD spectra file in the “Filepass” part of the code. The CD spectra data are contained in rows 20 to 1021 in the csv file obtained using a J1500 CD spectrometer. To read the data from row 20 to row 1021, “skiprows = 19” and “nrows = 1001” codes are used. Check the number of header lines to be skipped because the header style may depend on the version of the instrument. The read_csv function in Pandas encodes csv files in utf-8 by default. If you get a “UnicodeDecodeError” warning when reading the csv file, you need to explicitly specify the encoding along with the language included; see the list of supported encoding languages at the following link.
    https://docs.python.org/3/library/codecs.html#standard-encodings.
  h. Execute the following code to retrieve the wavelength from the read data.
    xlist = np.array(VEGF_IM_Um.iloc[:,0].drop(0))
    xlist
  i. Execute the following code to retrieve the temperature from the read data.
    temp_list = np.array(VEGF_IM_Um.iloc[0].drop(0))
    temp_list
  j. Execute the following code to retrieve the measured CD value from the read data.
    VEGF_IM_Um_CD = np.array(VEGF_IM_Um.drop(0,axis=0).drop(0,axis=1)).T
    VEGF_IM_Um_CD
    ***Note:*** In this protocol, the name is “VEGF_IM_Um_CD,” but any name can be specified.
  k. Execute the following code to confirm the matrix of the data.
    VEGF_IM_Um_CD.shape
  l. Execute the following code to display the CD spectra.
    for i in VEGF_IM_Um_CD:
     plt.plot(xlist, i)
  m. Execute the following code to retrieve the CD values at the positive peak.
    VEGF_IM_Um_290 = VEGF_IM_Um_CD[:,np.where(xlist==290)[0][0]]
    ***Note:*** The *VEGF* i-motif showed a positive peak at 290 nm; therefore, CD values at 290 nm were retrieved in this protocol. In this protocol, the name is “VEGF_IM_Um_290,” but any name can be specified.
  n. Execute the following code to display a scatter plot of CD value vs. temperature.
    plt.scatter(temp_list,VEGF_IM_Um_290, edgecolor='black', facecolor='white', s=100)
    plt.xlabel(r'Temperature /$ˆ\circ$C')
    plt.ylabel('CD / mdeg')
    ***Note:*** X and Y labels for graphs drawn with matplotlib can be written as strings or in LaTeX style.
  o. Execute the following code to define a two-state thermal denaturation model.
    def ThermalDenat(T, aN, bN, aD, bD, dH, Tm):
     T = T + 273.15
     Tm = Tm + 273.15
     R = 1.99
     Kd = np.exp((dH/R)∗(1/Tm - 1/T))
     theta = ((aN∗T+bN)+Kd∗(aD∗T+bD))/(1+Kd)
     return theta
  p. Execute the following code to define the X-axis, Y-axis, and initial parameters using nonlinear fitting.
    temp_list = temp_list
    yRaw = VEGF_IM_Um_290
    para_ini = [0, 20, 0, 5, 100000, 60]
    ***Note:*** To perform nonlinear fitting, it is necessary to provide initial values for each parameter. Here, para_ini = [$a_{N}$, $b_{N}\text{,}$$a_{D}$, $b_{D}$, ΔH, $T_{m}]$ is defined as an array of initial parameters.
  q. Execute the following code to fit the CD melting curves with the thermal denaturation model defined in step 17-o.
    para_opt, cov = optimize.curve_fit(ThermalDenat, temp_list,yRaw, para_ini)
    yFit = ThermalDenat(temp_list,
    para_opt[0],para_opt[1],para_opt[2],para_opt[3],para_opt[4], para_opt[5])
    dH = -para_opt[4]
    dH_SD = np.sqrt(np.diag(cov))[4]
    Tm = para_opt[5]
    Tm_SD = np.sqrt(np.diag(cov))[5]
    dS = (-para_opt[4]/(para_opt[5]+273.15))
    dS_SD = np.sqrt((dH_SD/dH)∗∗2 + ((dH/(Tm∗∗2))+Tm_SD)∗∗2)
    R2 = ((np.corrcoef(yFit, yRaw)[0][1])∗∗2)
    ***Note:*** The optimize.curve_fit function of SciPy uses the model equation, X-axis, Y-axis, and initial values of the parameters as arguments, and returns the optimized parameters and covariance matrix. The diagonals of the covariance matrix indicate the variance of the optimized parameters where R2 is the coefficient of determination. If you get a “RuntimeError” or “OptimizeWarning”, the appropriate optimization has not been performed. In this case, the 2-state denaturation model may not be appropriate (e.g., oligonucleotides denature through multiple states or are not fully denatured).
  r. Execute the following code to draw the recalculated melting curves using the optimized parameters and model equations.
    plt.figure(figsize=(5,3))
    plt.scatter(temp_list, yRaw, facecolor = 'white', edgecolor = 'black', s = 50,
       label='Raw')
    plt.plot(temp_list, yFit, linewidth = 2, color = 'red', label='Fit')
    plt.xlabel(r'Temperature /$ˆ\circ$C')
    plt.ylabel('CD/ mdeg')
    plt.title('Tm='+str(Tm.round(3))+ r'$ \pm$' + str(Tm_SD.round(3)) + '\n'+
       'dH='+str(dH.round(0))+ r'$ \pm$' + str(dH_SD.round(0)) + '\n'+
       'dS='+str(dS.round(0))+ r'$ \pm$' + str(dS_SD.round(0)) + '\n'+
       'R2='+str(R2.round(4)),
      loc='right')
    plt.legend()
    ***Note:*** Note that the signs of enthalpy (ΔH) and entropy (ΔS) are reversed for output as a folding parameter.
  s. Create a new project and repeat steps e-r for the analysis of the methylated CpG *VEGF* i-motif-forming oligonucleotides.

## Expected outcomes

**Figure 6:**
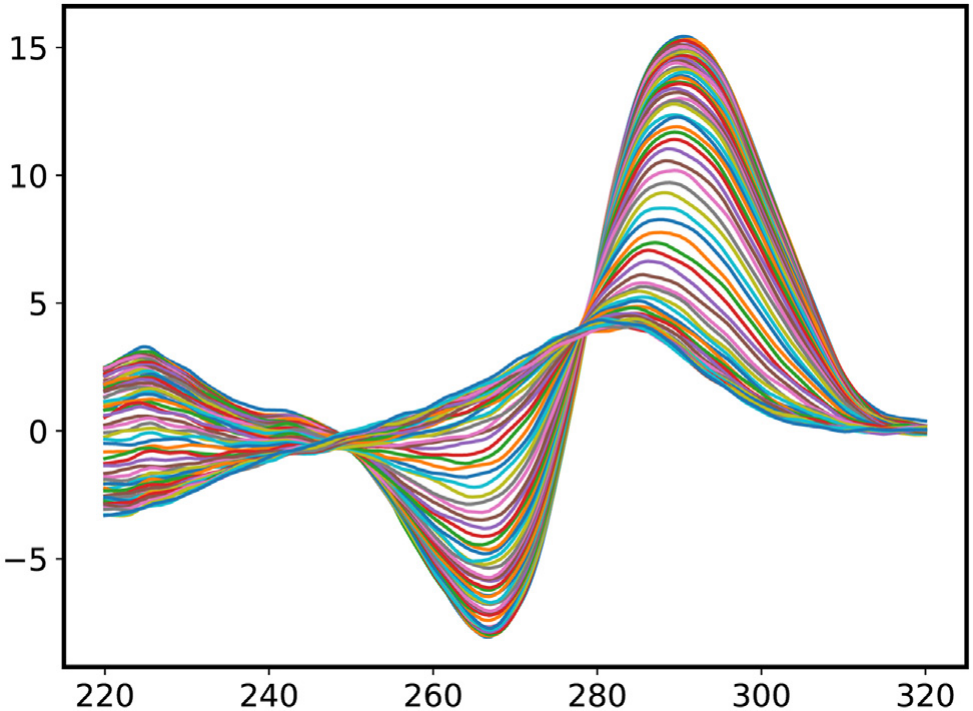
CD spectra of the unmethylated *VEGF* i-motif at 25°C–95°C obtained at step 17-I of the data analysis using Python 3

**Figure 7:**
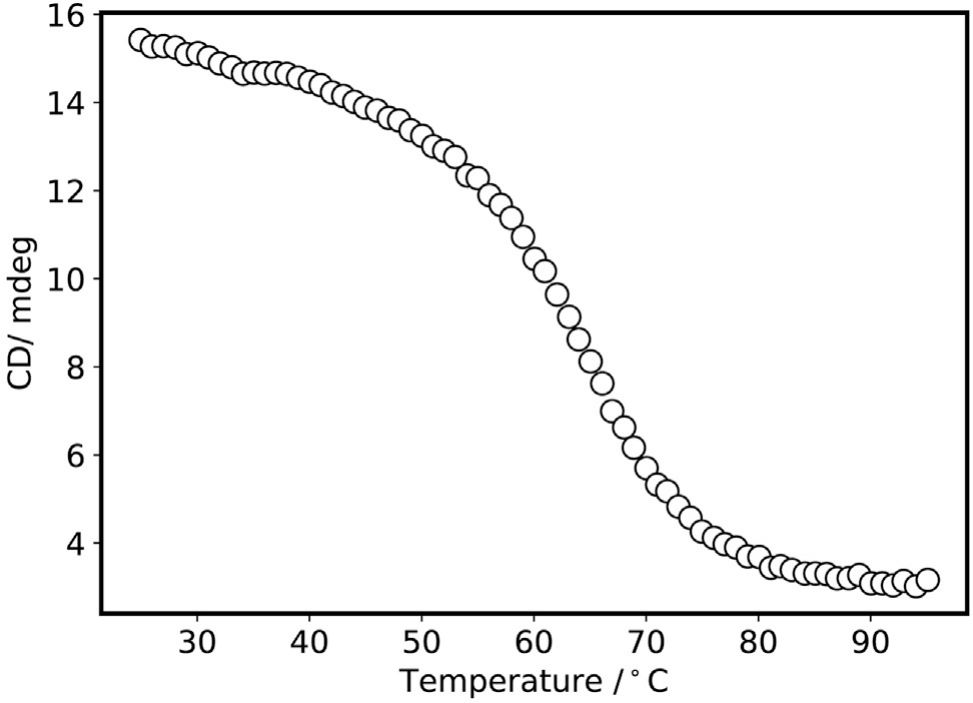
Scatter plot of CD value of the unmethylated *VEGF* i-motif vs. temperature obtained at step 17-n of the data analysis using Python 3

**Figure 8:**
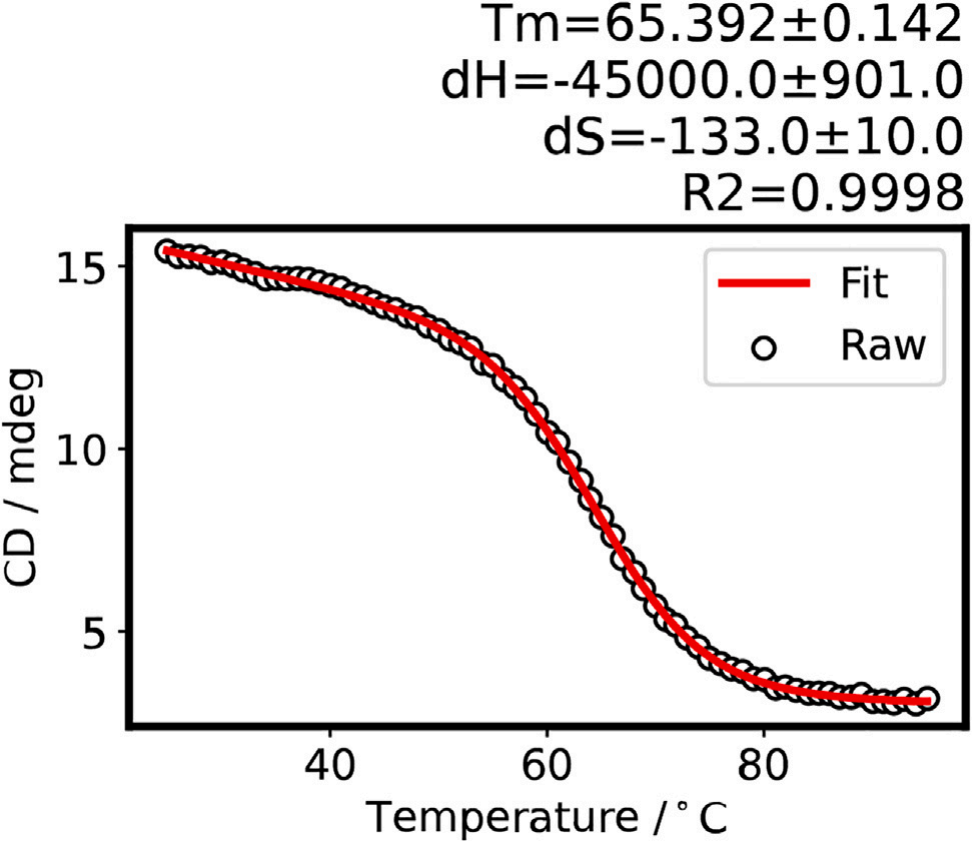
Melting curve and thermodynamic parameters for the unmethylated *VEGF* i-motif obtained at step 17-r of the data analysis using Python 3

**Figure 9:**
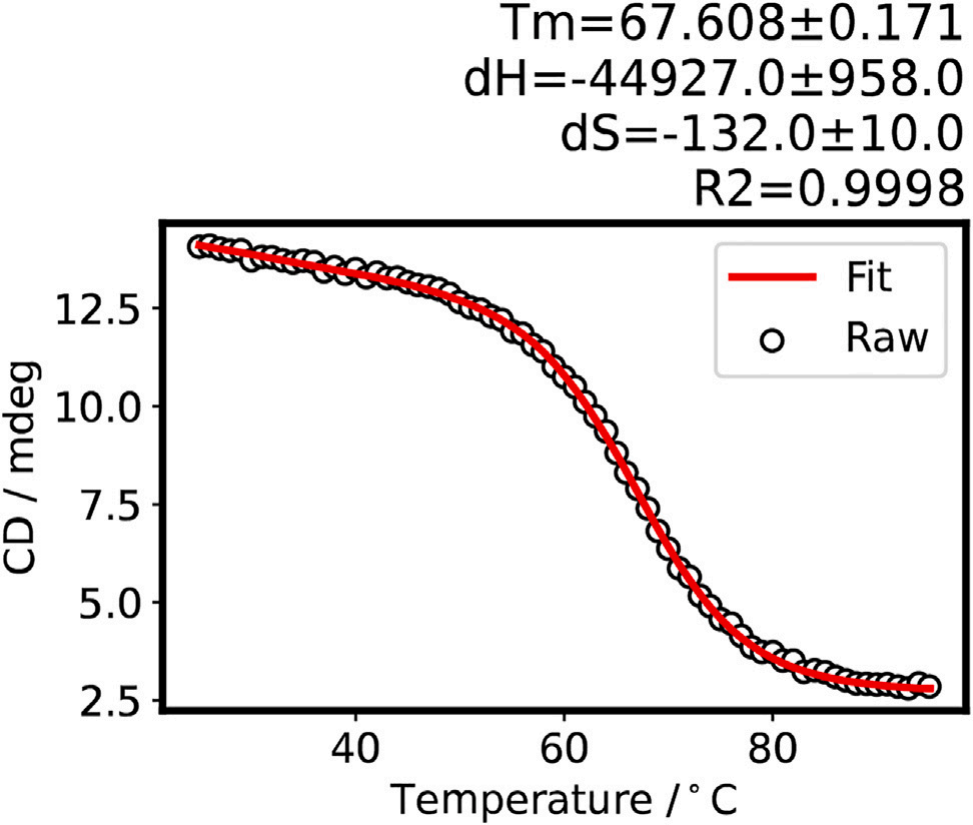
Melting curve and thermodynamic parameters for the methylated *VEGF* i-motif obtained at step 17-s of the data analysis using Python 3

This protocol was developed to measure the CD spectra of the G4/i motif structures and calculate the thermodynamic parameters using Python 3. In step 17-I of the data analysis using Python 3, a plot of all CD spectra at 25°C–95°C is obtained (Figure 6). In Step 17-n, a scatter plot of the CD value vs. temperature is obtained (Figure 7). In step 17-r and 17-s, the thermodynamic parameters ($T_{m}$, ΔH, and ΔS) of unmethylated and methylated *VEGF* i-motif structures are calculated (Figures 8 and 9).

## Limitations

This protocol cannot be applied to G4/i-motif structures in which thermal denaturation does not obey two-state thermal denaturation, for example, thermal denaturation of the human telomeric G4 structure is not a two-state thermal denaturation.^29^ In addition, G4/i-motif structures that are not fully denatured at 95°C cannot be analyzed.

## Troubleshooting

### Problem 1

Detection of large noise in the spectrum (related to Step 11, 14).

### Potential solution

Check that the HT (High-tension voltage), which is simultaneously measured with the CD, does not exceed 600 V. If it exceeds 600 V, reduce the sample or buffer concentration.

### Problem 2

Detection of step noise in the spectrum (related to Step 11, 14).

### Potential solution

Bubbles may have formed during CD measurements with increasing temperature. Degas the buffer before dissolving the sample. Alternatively, reduce the rate of temperature increase.

### Problem 3

Detection of step noise in the spectrum when measurements are started below 10°C (related to Steps 14).

### Potential solution

It may be a result of condensation on the cell surface, leave the cell in the cell holder for 30 min before starting the measurement.

### Problem 4

Observation of peak in blank buffer (related to Step 11).

### Potential solution

Quartz cells may have been contaminated. Add 1% Hellmanex to the cell and incubate for 2 h, then wash it with ultrapure water. If the cell is not clean, add concentrated nitric acid and incubate for 16 h, followed by washing with ultrapure water.

### Problem 5

Detection of a nonspecific CD spectrum of the G4/i-motif structure (related to Steps 9–15).

### Potential solution

The G4/i-motif-forming oligonucleotide may not form a G4/i-motif structure. Other techniques such as dimethyl sulfate footprinting, bromine footprinting, and nuclear magnetic resonance analysis should be performed to confirm the formation of G4/i-motif structures.^30^^,^^31^

## Resource availability

### Lead contact

Further information and requests for resources and reagents should be directed to and will be fulfilled by the lead contact, Wataru Yoshida (yoshidawtr@stf.teu.ac.jp).

### Technical contact

Technical questions on executing this protocol for CD measurement and thermodynamic parameters calculation should be directed to and will be answered by the technical contacts, Momo Moriya (moriya61m@gmail.com) and Taiji Oyama (taiji.oyama@jasco.co.jp), respectively.

### Materials availability

This study did not generate new unique reagents.

### Data and code availability

The published article includes all [datasets/code] generated or analyzed during this study.

## Acknowledgments

This work was supported by JSPS KAKENHI grant number 21K04798.

## Author contributions

M.M. and M.G. optimized and performed the CD spectra measurements. T.O. wrote the Python code and calculated thermodynamic parameters. K.I. and W.Y. supervised the study. W.Y. procured funding. M.M. and W.Y. wrote the manuscript. All authors have approved the final version.

## Declaration of interests

The authors declare no competing interests.

## Supplemental information

Data S1. Sample matrix data for measurement conditions, wavelengths in rows and temperatures in columns for unmethylated (VEGF_IM_Um.csv) VEGF i-motifs

Data S2. Sample matrix data for measurement conditions, wavelengths in rows and temperatures in columns for methylated (VEGF_IM_M.csv) VEGF i-motifs
